# Reassessing the association of *MUC5B* with survival in idiopathic pulmonary fibrosis

**DOI:** 10.1111/ahg.12522

**Published:** 2023-08-03

**Authors:** Siyang Cai, Richard J. Allen, Louise V. Wain, Frank Dudbridge

**Affiliations:** ^1^ Department of Population Health Sciences University of Leicester Leicester UK

**Keywords:** case‐only study, index event bias, instrumental variable, mendelian randomisation, selection bias

## Abstract

A variant in the mucin 5B gene (*MUC5B*) is strongly associated with the risk of idiopathic pulmonary fibrosis. However, the same variant is associated with increased survival time. Previous work suggested that this may be explained by index event bias, with the true effect being to decrease survival. Here, we reassessed this claim using more recent methods and datasets. We found that the statistical assumptions of the previous analysis did not hold, and instead, we applied recent methods of corrected weighted least squares, MR‐RAPS and Slope‐hunter to both the previous data and an updated consortium meta‐analysis. However, these analyses did not yield robust evidence for increased or decreased survival. In simulations of a true effect of decreased survival, we did not observe any realistic scenario in which index event bias led to an observed effect of increased survival. We therefore regard as unsafe the claim that *MUC5B* has a true effect of decreased survival. Alternative explanations should be sought to explain the observed association with increased survival.

## 1 INTRODUCTION

Idiopathic pulmonary fibrosis (IPF) is a progressive and terminal condition with substantial heritability. Genome‐wide association studies (GWAS) have identified a number of loci with effects on IPF (Allen et al., 2022), the strongest being in the promoter of the *MUC5B* gene with an odds ratio of approximately 5. With such a strong effect on risk, *MUC5B* is a natural target for functional studies to understand disease aetiology and identify potential treatments (Yang et al., 2015).

Surprisingly, the risk allele of *MUC5B* is associated with increased survival time, albeit more weakly than other loci (Peljto et al., 2013). This suggests that the gene may play a different role in disease progression to disease initiation, creating problems for designing and interpreting functional studies. A possible explanation is that *MUC5B* defines a subtype of IPF with better prognosis, although clinical or molecular characteristics of such a subtype have not been established (Yang et al., 2015). Another possible explanation is index event bias (Dahabreh & Kent, 2011). This is a statistical effect in which associations with a subsequent event (here survival) can be biased in studies that condition on an index event (presence of disease). If a case of IPF happens to carry the risk allele of *MUC5B*, then it is less likely to be carrying other risk factors. If those other factors also affect survival, then the *MUC5B* case will survive longer even if the gene has no effect itself. This bias can change the direction of an estimated effect; for example it has been suggested as a partial explanation for the association of obesity with improved survival in cardiovascular disease (Sperrin et al., 2016).

As common causes of index and subsequent events may be unknown, methods have been developed to adjust for index event bias using genome‐wide single‐nucleotide polymorphisms (SNPs) as instrumental variables (Cai et al., 2022). In the first application of these methods to IPF (Dudbridge et al., 2019), the hazard ratio for the *MUC5B* SNP rs35705950 was adjusted from 0.766 (95% confidence interval [CI]: 0.634–0.925) to 113.3 (95% CI: 11.58–126.6). Although this estimate is extraordinarily high and had considerable uncertainty, bootstrap analyses suggested that a hazard ratio less than 1 could be rejected at $P<10^{-4}$. Therefore, the association of *MUC5B* with increased survival could be explained by index event bias, with its true effect being to decrease survival.

However, this result depends upon the assumptions of the bias adjustment, and considering the substantial uncertainty in the adjusted hazard ratio, the result is far from conclusive. In particular, theory predicts low levels of index event bias in rare diseases (Yaghootkar et al., 2017), which should apply to IPF which has prevalence less than 63 per 100,000 (Nalysnyk et al., 2012). Here, we re‐examine the association of *MUC5B* with survival by further investigating the assumptions of the bias adjustment, applying recently improved methods and analysing a recent larger dataset.

## 2 MATERIALS AND METHODS

### 2.1 Data

Our previous analysis used the GWAS of Allen et al. (2017) consisting of 612 cases and 3366 controls with 7,983,997 SNPs genotyped or imputed. The bias adjustment requires a large set of independent and accurately genotyped SNPs, for comparison of their effects on index and subsequent traits (see below). Therefore, SNPs were filtered with imputation *R* ^2^ threshold of 0.99, and linkage disequilibrium pruning was performed using PLINK 1.90 in 250‐kb windows with *R* ^2^ threshold of 0.1. This yielded a set of 140,092 SNPs (Dudbridge et al., 2019) which we again used in the present study to re‐examine our previous result.

The largest and most recent GWAS of IPF is a meta‐analysis of five studies with 4125 cases and 20,464 controls in total (Allen et al., 2022). This includes the previous data together with other studies conducted in U.K., U.S. and Spanish populations. Survival data were available for 2668 cases (Oldham et al., 2023); these include all the research data currently available globally in the genetics of IPF survival. For both studies, GWAS summary statistics were used. We estimated the bias adjustment in this larger dataset using 134,576 SNPs from the previous set of 140,092 that were genotyped in at least four studies of the meta‐analysis.

### 2.2 Bias adjustment

Let *X* denote an index trait, such as incidence of disease, and *Y* a subsequent trait such as survival. Let *G* be a genotype, conventionally coded as the number of minor alleles carried at a SNP. Our interest is in the hazard ratio of genotype on survival, conditional on having the disease. Denoting the log hazard ratio by $\beta_{GY}$, it can be shown that (Dudbridge et al., 2019)

(1) $$\beta_{GY}\approx \beta_{GY}^{\prime }-b\beta_{GX},$$

where $\beta_{GY}^{\prime }$ is the log hazard ratio estimated in cases and $\beta_{GX}$ is the log odds ratio of genotype on disease. Here, *b* quantifies the bias as a function of $\beta_{GX}$, and it can be estimated as the slope of the regression of $\beta_{GY}^{\prime }$ on $\beta_{GX}$ across many independent SNPs. With the estimate of *b*, bias‐adjusted estimates of $\beta_{GY}$ are the regression residuals obtained from Equation (1). Of note, if the estimated log hazard ratio is negative and the log odds ratio is positive, as for *MUC5B*, then *b* must be negative for the adjusted log hazard ratio $\beta_{GY}$ to be positive.

This approach is derived under the assumption of no statistical interactions between any of *G*, *X* and unmeasured common causes of *X* and *Y*. Furthermore, the regression assumes that the effects on the index trait $\beta_{GX}$ are uncorrelated with the effects of interest $\beta_{GY}$. This may be assumed for all SNPs used in the study (Dudbridge et al., 2019), or a subset may be identified for which the assumption is more plausible (Mahmoud et al., 2022). Violation of these assumptions may lead to an over‐ or under‐correction for index event bias.

We first followed Dudbridge et al. (2019) by estimating *b* from ordinary least squares regression of SNP log hazard ratios for survival on their log odds ratios for IPF. To account for possible violation of linear regression assumptions, we repeated the analysis with an inverse variance‐weighted regression, where the variances are the squared standard errors of the log hazard ratios (Cai et al., 2022). We further estimated *b* using the Slope‐hunter algorithm (Mahmoud et al., 2022) with default parameters, and with MR‐RAPS (Zhao et al., 2020) using the analogy between index event bias adjustment and Mendelian randomisation (Cai et al., 2022). The analyses were performed in both the previous and most recent datasets.

### 2.3 Simulation

We performed a simulation aiming to reproduce the observed hazard ratio for survival under the assumptions of the bias adjustment. For individual *i*, we simulated genotype $G_{i}$ with additive coding under Hardy–Weinberg equilibrium with minor allele frequency 0.11, similar to rs35705950 in *MUC5B* in European‐ancestry populations. We calculated disease risk $p_{i}$ as

$$\log\left(\frac{p_{i}}{1-p_{i}}\right)=\beta_{0}+\beta_{GX}G_{i}+\beta_{UX}U_{i},$$

where $\beta_{GX}=\log\left(5\right)$ similar to rs35705950, $U_{i}$ was simulated from a standard normal distribution and $\beta_{UX}$ was varied from 0 to 20. Here, $U_{i}$ represents the combination of all risk factors other than the *MUC5B* genotype. For each value of $\beta_{UX}$, the value of β_0_ was numerically solved such that the mean risk $p_{i}$ was 0.05%. We simulated 2 million individuals so that 1000 cases of disease occurred on average.

For the cases, survival times were then simulated under the proportional hazards model using the inverse probability method with Weibull baseline hazard (Bender et al., 2005). For individual *i*,

$$t_{i}={\left(\frac{-\log\left(v_{i}\right)}{\lambda \exp\left(U_{i}\right)}\right)}^{\frac{1}{\rho }},$$

where $v_{i}∼\mathrm{Unif}\left(0,1\right)$, λ=exp(−12.5) and ρ=1.7 to approximate the distribution of survival times in the IPF dataset. Note that *U* affects both disease risk and survival. There was no effect of genotype on survival, so the estimated effect equals the index event bias due to *U*. This was obtained using Cox regression with *G* as the sole predictor and the mean bias estimated from 1000 simulations. As the observed log hazard ratio of log(0.766) is the unbiased estimate plus a bias, we subtracted the estimated bias from log(0.766) to estimate the true hazard ratio.

## 3 RESULTS

### 3.1 Bias adjustment

In the previous data, we estimated the slope *b* in Equation (1) by the regression of log hazard ratios for survival on log odds ratios for risk. This must be negative if the true hazard ratio of *MUC5B* is greater than 1 (Equation 1). This was the case in the unweighted regression, where the estimate of *b* was −0.025 (95% CI: −0.0317 to −0.0183; *p* = 3 × 10^−13^), which is the result previously reported (Dudbridge et al., 2019). However, the weighted regression gives an estimate of −0.001 (95% CI: −0.0074 to 0.0055; *p* = 0.77), with a CI including positive values.

**FIGURE 1:**
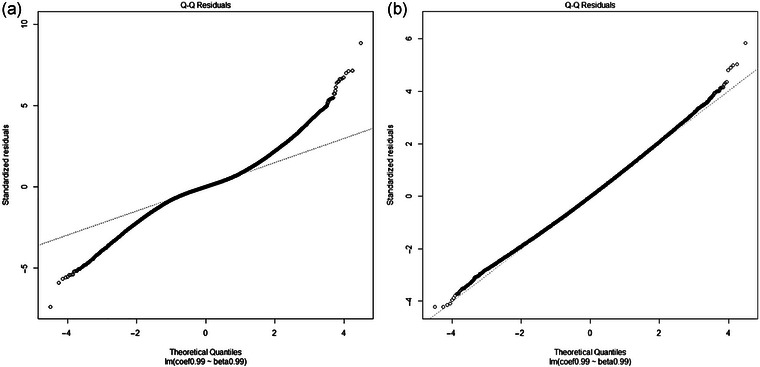
Normal quantile–quantile plots for the linear regression of log hazard ratios for survival on log odds ratios for IPF risk. (a) Unweighted regression. (b) Inverse variance weighted regression.

**FIGURE 2:**
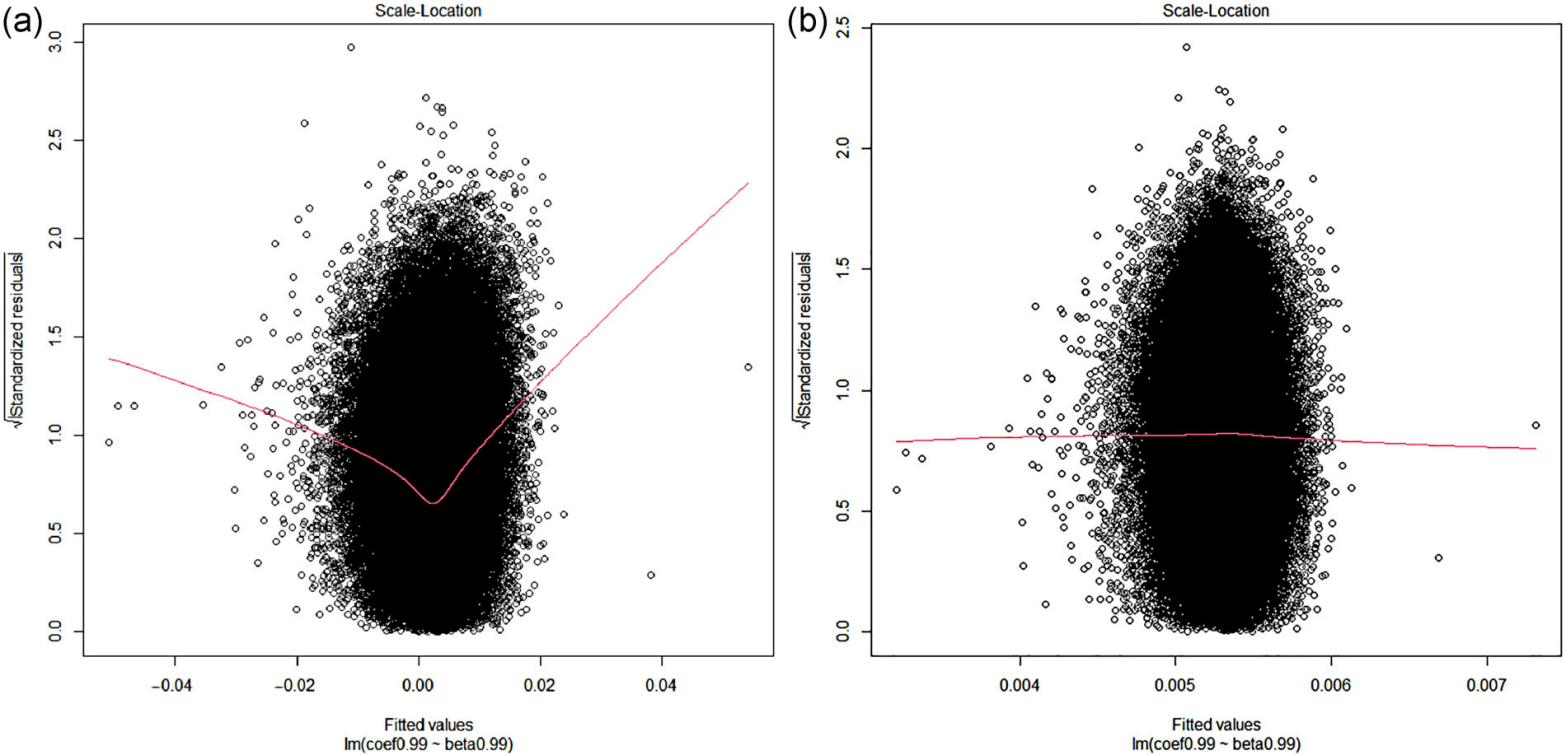
Scale–location plots for the linear regression of log hazard ratios for survival on log odds ratios for IPF risk. (a) Unweighted regression. (b) Inverse variance weighted regression.

In Figures 1 and 2, we check the assumptions of the linear regressions using diagnostic plots. Figure 1 shows the normal quantile–quantile plot, which is used to examine whether the residuals are normally distributed. It is obvious that the residuals do not follow the straight reference line for the unweighted regression, but the fit is better for the weighted regression. Figure 2 shows the scale–location plot, which checks the homogeneity of variance. A horizontal red line represents an equal spread of residuals along the range of predictors. Again, this assumption seems to be violated in the unweighted regression but not in the weighted regression. Therefore, the diagnostic plots suggest that the weighted regression is the more reliable.

**FIGURE 3:**
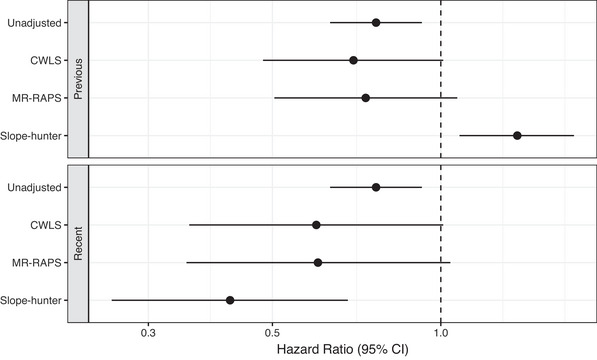
Hazard ratios for survival for rs35705950, without (unadjusted) and with adjustment for index event bias. Previous data consist of 612 cases and 3366 controls in which the odds ratio for IPF risk is 5.64 (95% confidence interval [CI]: 4.73−6.72). Recent data consist of 4125 cases and 20,464 controls in which the odds ratio for IPF risk is 5.06 (95% CI: 4.69−5.47).

The regression estimates of *b* are biased towards zero because of sampling variation in the log odds ratios (Dudbridge et al., 2019). We have proposed a corrected weighted least squares (CWLS) estimator to adjust *b* for this bias (Cai et al., 2022). Figure 3 shows adjusted hazard ratios for rs35705950 using CWLS, MR‐RAPS (Zhao et al., 2020), and Slope‐hunter (Mahmoud et al., 2022). In the previous data, CWLS and MR‐RAPS gave similar results with hazard ratios remaining below 1 but 95% CIs that included 1. However, Slope‐hunter gave an adjusted hazard ratio whose CI was entirely greater than 1.

We applied these methods to the most recent data (Allen et al., 2022; Oldham et al., 2023), which expand the previous data from 612 cases to 4125 (survival data for 2688) and from 3366 controls to 20,464 (Figure 3). Now the adjusted hazard ratio from Slope‐hunter is also less than 1, while the CIs for both CWLS and MR‐RAPS still include 1. Although the Slope‐hunter results are not consistent between the two datasets, the salient point is that we do not observe robust evidence that *MUC5B* has an effect on either increased or decreased survival.

### 3.2 Simulation

**FIGURE 4:**
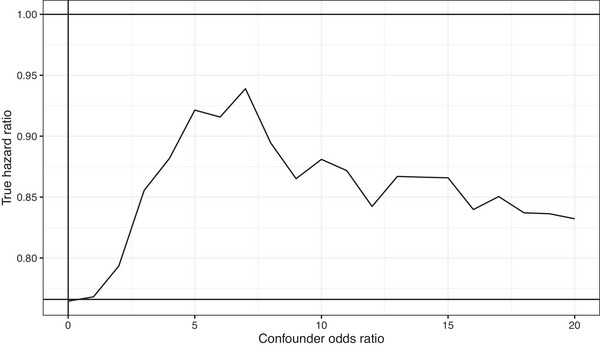
True hazard ratio yielding an observed hazard ratio of 0.766 under index event bias due to common causes of risk and survival. Confounder odds ratio is the effect of such common causes on disease risk.

The simulation was based on the prevalence of IPF and survival times in the U.K. population. The confounder log odds ratio $\beta_{UX}$ was varied to induce different degrees of index event bias. As described in Section 2, we estimated the true log hazard ratio that would yield the observed value of 0.766. Figure 4 shows that the true hazard ratio remains less than 1 even in the scenario with the most bias. Therefore, under the models used in the data analysis (logistic regression for risk, and Cox regression for survival), we could not identify a scenario in which the observed hazard ratio less than 1 was a biased estimate of a true hazard ratio greater than 1.

## 4 DISCUSSION/CONCLUSION

Knowledge of whether *MUC5B* has a beneficial effect on survival could inform the subtyping of IPF and guide mechanistic studies. Previously, we developed a method using genome‐wide SNPs to estimate the impact of index event bias on estimates of the effect of the *MUC5B* SNP on survival. That previous study suggested that there was index event bias and that the true effect of the MUC5B SNP was to decrease survival, although the adjusted hazard ratio had substantial uncertainty. In the present study, with refinements to the method, we show that the previous inference of decreased survival is unsafe, and the direction of the association remains uncertain.

We re‐examine the result by looking more closely at the assumptions of the method, applying recent methods (Cai et al., 2022) and analysing more recent data (Allen et al., 2022; Oldham et al., 2023). We find that the assumptions of unweighted linear regression are violated, and so the previous result is unreliable. Using inverse variance weighting, the diagnostic plots are more reasonable but minimal evidence for index event bias is observed. The adjusted hazard ratios remained less than 1 using the recent approaches of CWLS and MR‐RAPS but with CIs that included 1. Slope‐hunter gave an adjusted hazard ratio greater than 1 in the previous data, but this was reversed in the most recent data. In simulations, we found that the observed hazard ratio of 0.766 could not arise from a true hazard ratio greater than 1, even when a high level of index event bias was assumed.

In summary, when using more appropriate methods, we did not find robust evidence for index event bias nor for the association of *MUC5B* with either increased or decreased survival. It is still uncertain whether the observed association with increased survival is robust, but we regard as unsafe the previous result suggesting an association with decreased survival. It is possible that larger studies will provide more accurate estimates of the adjusted hazard ratio. However, alternative explanations should be sought for the association of *MUC5B* with IPF survival.

## AUTHOR CONTRIBUTIONS

Siyang Cai analysed data, implemented software, interpreted results, and drafted the manuscript. Richard J. Allen implemented software, provided data, interpreted results, and reviewed the manuscript. Louise V. Wain conceived the study, interpreted results, and reviewed the manuscript. Frank Dudbridge conceived the study, implemented software, interpreted results, and drafted the manuscript.

## CONFLICT OF INTEREST STATEMENT

The authors declare no conflicts of interest.

## Data Availability

Summary statistics for IPF risk and survival are available via https://github.com/genomicsITER/PFgenetics.
